# Microbial Antigen Identification and Vaccine Delivery Systems

**DOI:** 10.3390/vaccines10122053

**Published:** 2022-11-30

**Authors:** Paulo J. G. Bettencourt, Jorge H. Leitão

**Affiliations:** 1 Faculty of Medicine, Universidade Católica Portuguesa, 2635-631 Rio de Mouro, Portugal; 2 Centre for Interdisciplinary Research in Health, Universidade Católica Portuguesa, 1649-023 Lisboa, Portugal; 3 Department of Bioengineering, IBB—Institute for Bioengineering and Biosciences, Instituto Superior Técnico, Universidade de Lisboa, Av. Rovisco Pais, 1049-001 Lisboa, Portugal; 4 Associate Laboratory, i4HB—Institute for Health and Bioeconomy at Instituto Superior Técnico, Universidade de Lisboa, Av. Rovisco Pais, 1049-001 Lisboa, Portugal

Vaccine efficacy and immunogenicity depend on the host, pathogen, and pathogenesis of the disease. The optimization of vaccine efficacy is therefore a requirement of many vaccines, including approved ones. Key aspects of vaccine design and development involve the identification of the foremost immunogenic determinants of a pathogen and the choice of the best delivery system. Different vaccine delivery systems, using the same antigen, can induce variable outcomes. Therefore, the selection and evaluation of the ideal delivery system are paramount for the success of a vaccine. Recent advances in the identification of immunogenic determinants have been achieved, with impressive developments, as is the case for immunopeptidomics. A wide range of delivery systems have been developed so far, from variolation in ancient China dating back to the 15th century to the most advanced viral vectors and RNA vaccines currently used for the development of COVID-19 vaccines in the 21st century [1,2]. In this Special Issue, we present articles focused on innovative methods for the identification of immunogenic determinants from microbial pathogens, irrespective of their biochemical nature or antigen presentation pathway. Given that the efficacy of a vaccine antigen depends greatly on the delivery method, this Special Issue also focuses on improving microbial-based delivery systems.

The *Mycobacterium bovis* strain Bacillus Calmette Guerin (BCG), is the oldest approved vaccine in the world and is still in use [2,3]. Using the host–pathogen interaction model of THP-1 macrophages infected with BCG, Jamie Medley and colleagues describe a very robust strategy to identify the genetic adaptation of mycobacteria to the intracellular environment. The authors identified 329 significantly differentially regulated genes, 24 h after infection. This research article generated important RNAseq datasets on the BCG response to human macrophages, which can bring important new vaccine antigens and drug targets [4]. Another type of intracellular bacterium that has been causing health threats, mostly in low-income countries, is the Gram-negative microorganisms of the *Rickettsiae* genus. Anke Osterloh presents a review of the current knowledge on immunology and immunopathology in Rickettsial infections, with focus on experimental vaccination against Rickettsial diseases [5]. The fight against extracellular bacteria and their toxins can benefit from the recent developments in antibody technology. António Seixas and colleagues review mammalian polyclonal and monoclonal antibodies, avian immunoglobulin Y antibodies, and single-domain antibodies, and discuss their potential for the development of antibody-based immunotherapies against bacteria [6].

The intracellular pathogens studied in this Special Issue cause serious health concerns, have been developing antibiotic-resistant strains, and require new vaccines. The methods and technologies described here may contribute to improved vaccine design and development. We hope that this Special Issue will contribute to stimulating future research in the development of vaccines against intracellular and hard-to-treat bacterial diseases.
